# Venom Components of Iranian Scorpion *Hemiscorpius lepturus* Inhibit the Growth and Replication of Human Immunodeficiency Virus 1 (HIV-1)

**DOI:** 10.22045/ibj.2016.02

**Published:** 2016-11

**Authors:** Rezvan Zabihollahi, Kamran Pooshang Bagheri, Zohreh Keshavarz, Fatemeh Motevalli, Golnaz Bahramali, Seyed Davar Siadat, Seyed Bahman Momen, Delavar Shahbazzadeh, Mohammad Reza Aghasadeghi

**Affiliations:** 1 Department of Hepatitis and AIDS, Pasteur Institute of Iran, Tehran, Iran; 2 Biotechnology Research Center, Pasteur Institute of Iran, Tehran, Iran; 3 School of Nursing and Midwifery, Shahid Beheshti University of Medical Sciences, Tehran, Iran; 4 Department of Microbiology; Pasteur Institute of Iran; Tehran, Iran; 5 Department of Pilot Nano-Biotechnology, Pasteur institute of Iran, Tehran, Iran

**Keywords:** Human immunodeficiency virus (HIV), Herpes simplex virus (HSV), Hemiscorpius lepturus, Venom

## Abstract

**Background:**

During the recent years, significant progress has been achieved on development of novel anti-viral drugs. Natural products are assumed as the potential sources of novel anti-viral drugs; therefore, there are some previous studies reporting the anti-viral compounds from venomous animals. Based on the significant value for tracing of non-toxic anti-viral agents from natural resources, this study was aimed to investigate the anti-viral activity of some HPLC purified fractions derived from the venom of Iranian scorpion, *Hemiscorpius lepturus*, against human immunodeficiency virus 1 (HIV-1) and herpes simplex virus 1 (HSV-1).

**Methods:**

*H. Lepturus* crude venom was subjected to reverse phase HPLC analysis to determine its active components precisely where four dominant fractions obtained at retention time of 156-160 minutes. The phospholipase A2 and hemolytic activities of the purified fractions were first evaluated. Then the anti-viral activity was measured using single cycle HIV (NL4-3) replication and HSV (KOS) plaque reduction assays.

**Results:**

The *H. lepturus* crude venom inhibited HIV replication by 73% at the concentration of 200 µg/ml, while it did not show significant anti-HSV activity. It also inhibited the cell-free viral particles in a virucidal assay, while it showed no toxicity for the target cells in a proliferation assay. The four HPLC fractions purified from *H. lepturus* inhibited HIV with IC_50_ of 20 µg/ml.

**Conclusion:**

H. *lepturus* venom contains components with considerable anti-HIV activity insofar as it has virucidal activity that offers a novel therapeutic approach against HIV infection. Our results suggest a promising pilot for anti-HIV drug discovery with *H. lepturus* scorpion venom.

## INTRODUCTION

Since immemorial times, animal venoms have been used as potent drugs to cure different disease states[1]. In traditional medical practice, scorpion venoms have been applied for the treatment of various ailments, such as acute and chronic convulsion, epilepsy, subcutaneous nodules and tetanus for more than 2100 years[2]. As written information on Chinese herb states, snake and scorpion venoms contain many different biologically active proteins and peptides that have pharmacological activities such as anti-microbial[3], anti-epileptic[4], and channel-blocking activities[5,6]. Recently, some possible clinical usages of venoms have also been described[7,8].

*Hemiscorpius lepturus* is an Iranian scorpion found in southwest of Iran, and its venom contains hemolytic, proteolytic, cytotoxic, anti-cancer and channel blocker compounds with possible therapeutic potency[9-13]. Despite the significant success of highly anti-retroviral therapy, AIDS is still one of the urgent world health problems [14]. Although conventional drugs targeting viral components of HIV are widely being used and have shown satisfactory results [15,16], there is a growing demand for the discovery of new therapeutic agents with either natural or synthetic origins. Herpes simplex virus (HSV) is another common virus that causes different types of diseases, with different degrees of severity ranging from mild to severe. In certain cases, it may possibly lead to life-threatening situation, particularly in immune-compromised patients. After the primary infection, HSV establishes itself by permanently residing in the ganglia of the host neurons. Nucleoside analogs as well as acyclovir and other nucleoside derivatives, including famciclovir, valaciclovir, and penciclovir have been approved for the treatment of HSV infections[17]. However, the formation of acyclovir-resistant HSV is an increasing problem.

In the last decade, natural derivatives have been considerably used as the popular remedies to prevent or manage the chronic diseases, to improve cognitive function and to increase longevity[18]. Natural medicines have reportedly important pharmacological activities and are capable of producing therapeutic effects[19,20]. A large number of natural agents have already been used as anti-viral products, and for some of these compounds, the target molecules and mechanism of action have been identified. A previous study has described a novel peptide, called p3bv, which had been extracted from the bee venom and inhibited T-tropic, but not M-tropic HIV, through its interaction with CXCR4[21]. It has also been reported that the snake venom administration could decrease the viral load and increase CD4 cells count for one year in a patient with multidrug-resistant HIV, high viral load, and low CD4 count[22,23].

In the field of venomics, emergency medicine has traditionally been most concerned about envenomation syndromes, their treatment and investigative therapeutic strategies of envenomation. Taking an alternative viewpoint, here in this paper, we have evaluated the anti-viral activity of venom from *H. lepturus* to inhibit the replication of HIV and HSV virions. We also investigated the anti-viral activity and the cytotoxicity of the crude venom and its purified fractions with phospholipase activity. Furthermore, the virucidal activity of the above mentioned venom was measured to identify its possible mechanism of action.

## MATERIALS AND METHODS

### Ethics statement

All animal experiments, including maintenance, animals’ handling program, and sample collection were approved by Institutional Animal Care and Research Advisory Committee of Pasteur Institute of Iran (Research Deputy, dated October 2010), based on the Specific National Ethical Guidelines for Biochemical Research issued in 2005 by the Research and Technology Deputy of Ministry of Health and Medicinal Education (MOHM) of Iran.

### Venom preparations

Adult *H. lepturus* scorpions were collected from Khuzestan and housed in well-ventilated wooden cages with food and water, supplied *ad libitum*. Venom was collected by electrical stimulation of the telson (8 volt), and the collected samples were pooled. The collected venom was lyophilized and then stored at -20°C[24].

### Purification

The *H. lepturus* lyophilized venom was weighed, and its protein concentration was measured by Bradford’s method[25]. Crude venom (6 mg) was dissolved in 0.05% trifluoroacetic acid in HPLC grade water and centrifuged at 2×10^4^ g or 10 min. The clear supernatant was used for purification. Reverse phase HPLC was performed in a HPLC system (Knauer GmbH, Germany) at a flow rate of 1 ml/min for 180 min. The fractions were separated by C18 column (250 mm×4.6 mm), 5 μm particle size, 100Ǻ pore size) with a linear gradient of 0.1% trifluoroacetic acid in water (solution A) and acetonitrile (solution B) 5% for 10 min, followed by 5-15% over 20 min, 15-45% over 120 min, and 45-78% over 20 min. The protein fractions were detected at 214 nm, and collected manually, dried in a vacuum concentrator and stored at -20°C.

### Phospholipase activity assay

Serial amounts of the isolated fractions and venom prepared in 100 µL deionized water. Then the substrate solution (100 µL containing lecithin, ethanol, Triton X-100, NaCl, Phenol red, CaCl_2_ and H_2_O) was added to each well and incubated at 37°C for 15 min. Deionized water was used as negative control and phospholipase A2 from Macrovipera lebetina snake venom was used as positive control. Optical density was documented at 550 nm in a microplate spectrophotometer (EPOCH, BioTeK, USA).

### Cells and transfection

Hela, HEK293T, and Vero cells were obtained from the Cell Bank of Pasteur Institute of Iran (Tehran) and maintained in DMEM medium (Gibco, Germany) containing 10% fetal calf serum (Biosera, Iran), 50 U/ml penicillin (Gibco, Germany), and 50 mg/ml streptomycin (Gibco, Germany). Plasmids were transfected into HEK293T cells using Polyfect transfection reagent (Qiagen, USA) according to the manual instruction. Briefly, 3.7×10^5^ HEK293T cells were seeded in 6-wells plates and transfected after 24 h. HEPES at the concentration of 25 nM was added to the culture medium during the transfection.

### Viruses

Single cycle replicable (SCR) HIV-1 virions pseudotyped with vesicular stomatitis virus surface glycoprotein were produced using transfection[14,26]. A plasmid mixture of pSPAX2, pMD2G (Addgene, Germany), and pmzNL4-3 (patented vector of Pasteur Institute of Iran) was used for each well, and DNA-transfection complex was removed after 8 h[14,27,28]. The virions were harvested at 24, 48, and 72 h post transfection, pooled and kept at 4°C. The pooled supernatants were clarified by 5-min centrifugation at 2×10^4^ g and filtered through 0.45-µm filters. The viruses were stored at -80ºC and were then evaluated for their infectious titers[29,30].

HSV-1 (KOS strain) was used in this study for the assessment of anti-HSV activity. To prepare the HSV virus stock, Vero cells (4.5×10^5^) were seeded in 6-well plates and infected by HSV virions after 24 h. The cell supernatants were harvested every 24 h after the infection until 96 h. The harvested supernatants were pooled and then clarified using 0.45-µm filters. The virus stock was stored at -80ºC and tittered for plaque-forming activity.

### HIV replication assay

Hela cells were placed in 96-wells plates (8×10^3^ cells per well) and used as targets of infection. The venom was added into the culture medium 2 h prior to the infection, and its concentration was maintained for 72 h after the infection. The cells were infected with 300 ng P24 SCR HIV and then incubated for 24 h to absorb the virions. The infected Hela cells were washed twice with phosphate buffered saline 24 h after infection and fed with 200 µl fresh medium. The plates were incubated for an additional 48 h and then centrifuged at 3.5×10^3^ g for 15 min. The supernatants were analyzed by P24 capture ELISA (Biomerieux, France). The minimal concentration of the venom required to suppress the load of P24 by 50% (IC_50_) was determined by regression analysis of the dose response curve.

### HSV plaque reduction assay

The HSV inhibitory effect was evaluated by plaque reduction assay. Vero cells (4×10^5^) were seeded onto 24-well plates and incubated for 24 h to reach at least to 96% confluency. The Vero cell monolayer was infected with 50 pfu of HSV-1 (KOS) and after 1 h, it was washed with pre-warmed DMEM and overlaid with methylcellulose (1.2%). The plates were further incubated in the presence of 5% CO_2_ at 37ºC for 72 h. Later, the overlay medium was aspirated, and the cell monolayer was precisely washed. Plates were fixed with methanol and then stained with 0.5% crystal violet. The inhibition percentage of HSV infection was determined by considering the reduction in plaques number.

### Virucidal effect

The direct inhibitory activity of the venom on HIV particles infectivity was evaluated by virucidal effect assay. The venom (200 µg/ml) was mixed thoroughly with 1.1×10^3^ P24 of HIV in a final volume of 11 µl. The mixture was incubated at 37ºC for 2.5 h, and then the reaction was stopped by adding 80 µl fresh complete medium. Residual virus infectivity was evaluated by HIV replication assays as mentioned above. The higher percentage of viral infectivity indicates the lower virucidal efficacy of the venom.

### Cellular toxicity

The cytotoxicity of the venom was tested by XTT (sodium 3’-[1-(phenylaminocarbonyl)-3,4-tetrazolium]-bis (4-methoxy-6-nitro) benzene sulfonic acid assay (Roche, Germany) according to the manufacture’s instruction. Hela cells (10^4^ in each well of a 96-wells plate) were treated with the venom for 72 h. After that, the medium was aspirated and fresh phenol red-free DMEM was added into the wells. Next, XTT solution was added (50 μl) into each well and then plates were incubated for 3 h. The absorbance of the sample was evaluated at 450 nm with a reference wavelength of 630 nm, and the CC_50_ (50% cytotoxicity concentration) was estimated from the plots.

## RESULTS

### Anti-viral potential of the crude venom

**Fig. 1:**
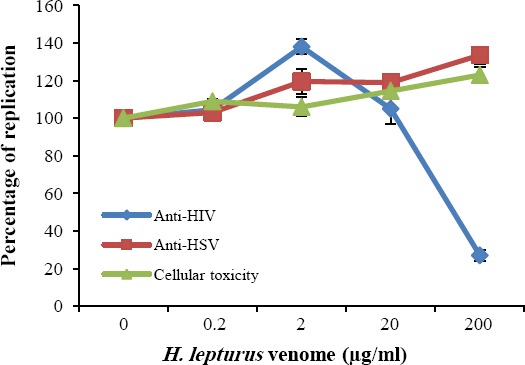
Does response activity. The anti-viral and cellular toxicity of *H. lepturus* venom for Hela cells. The HIV replication and HSV plaque reduction assays were used to investigate the anti-viral activity. The cytotoxicity was determined by XTT proliferation assay. The *H. lepturus* did not show anti-HSV and cytotoxic effect, while it indicated remarkable inhibitory activity against HIV, especially in higher concentrations.

The crude venom of *H. lepturus* was assessed for its anti-viral activity against HIV and HSV. The virions were cultured in the presence of 0.2, 2, 20, and 200 µg/ml concentrations of the venom, and the inhibitory effect was monitored by HIV replication and HSV plaque reduction assays. As shown in Figure 1, *H. lepturus* inhibited 73% infection at 200 µg/ml but did not show any significant activity in lower concentrations. The crude venom slightly raised the HSV replication at concentrations of more than 20 mg/ml, while it did not demonstrate any significant toxicity for the target Hela cells (Fig. 1).

### Virucidal effect of the venom

The neutralizing effect of *H. lepturus* venom against HIV particles was investigated by virucidal assay. The virucidal activity of the crude venom was consistent with its anti-replication activity. The replication capacities of HIV and HSV virions were evaluated after incubation with *H. lepturus* venom for 2.5 h. The *H. lepturus* crude venom showed significant activity for neutralizing the HIV particles; however, it did not have any significant effect on HSV virions. Furthermore, the venom neutralized 60.5% of HIV virions at 200 µg/ml, and no remarkable anti-HIV activity was observed in lower concentrations (Table 1).

**Table 1:** The neutralizing effect of *H. lepturus* venom against HIV and HSV particles

Concentration (mg/ml)	Percentage control of viral infectivity (%)	

	HIV-1	HSV-1
0.2	101.1±3.6	100.5±8.5
2	97.9±2.8	103.2±8.4
20	87.2±1.9	94.7±14.6
200	39.5±5.3	83.2±5.5

### The activity of extracted phospholipases from *H. lepturus* venom

**Fig. 2:**
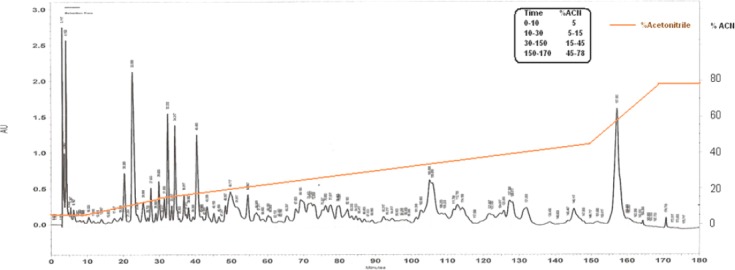
HPLC for *H. lepturus* crude venom.

**Fig. 3:**
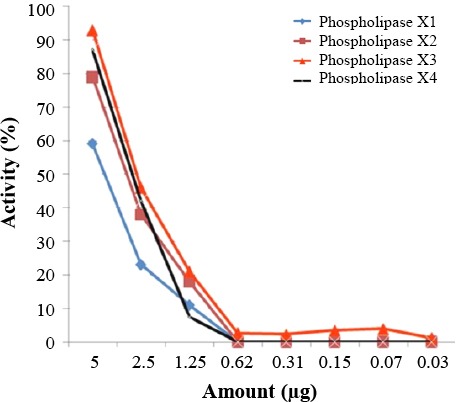
Phospholipase activity of purified fractions.

**Fig. 4:**
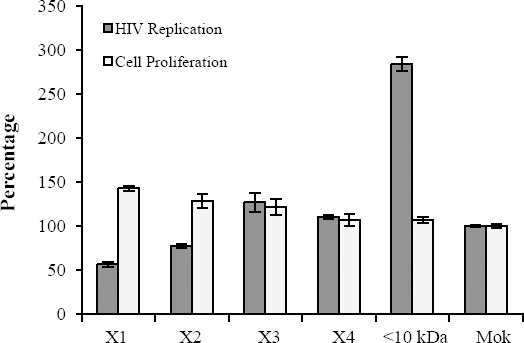
Anti-HIV activity of various fractions of *H. lepturus* venom. One of the extracted phospholipases (X1) showed the most anti-HIV activity among fractions extracted from crude venom. It inhibited about 43.5% of replication at 20 µg/ml concentration. The *H. lepturus* venome also encompassed a fraction of small peptides (>10 kDa) with considerable activity for facilitating HIV replication.

To determine the active components of *H. lepturus* venom, the venom was fractionated using HPLC system. For this purpose, over 90 fractions were purified from *H. lepturus* venom. Hemolytic fractions were separated from HPLC column at retention time of 155-158 minutes and acetonitrile concentration between 45 and 78% (Fig. 2). The extracted phospholipases were investigated for their hemolytic activity. Hemolytic activity of phospholipase X1, X2, X3, and X4 at concentration 5 µg/µl were 59, 78, 92, and 87%, respectively (Fig. 3). The anti-HIV potential of the fractions was evaluated by replication and cytotoxicity assays. The X1 and X2 phospholipases inhibited the HIV replication by 43.5 and 22.5%, respectively (Fig. 4). The *H. lepturus* venom interestingly encompassed a fraction of peptides with less than 10 kDa, which showed a high potential for facilitating HIV replication, inasmuch as the P24 load was increased approximately three-folds in the presence of the mentioned fraction (20 µg/ml).

## DISCUSSION

In the current study, the anti-viral property of scorpion *H. lepturus* venom was evaluated by HIV replication and HSV plaque reduction assays, while its cytotoxicity was monitored by XTT proliferation assay. *H. lepturus* venom showed negligible cytotoxicity for Hela cells and had specific impact on the viral replication. *H. lepturus* crude venom was shown to be a significant inhibitor against HIV virions (Fig. 1). This venom seems to affect the HIV but not the HSV replication, which demonstrates its specific interference in HIV life cycle. The analysis of the dose-response curve indicated the difference between the anti-viral effects of *H. lepturus* at concentrations lower and higher than 20 µg/ml (Fig. 2).

*H. lepturus* venom slightly raised the replication of HIV at 2 µg/ml whereas at higher concentrations (200 µg/ml), it notably inhibited the virions. The unique activity of *H. lepturus* against HIV replication, but not HSV, indicates that the venom of this scorpion may contain anti-HIV active components. The mechanism of anti-HIV activity was investigated by evaluating the direct effect of the venom on HIV particles. The virucidal effect of *H. lepturus* venom was similar to its inhibitory effect against HIV and HSV replication (Table 1). These data verified the anti-HIV potential of *H. lepturus* venom and depicted a possible mode of its anti-HIV action. The virucidal activity highlights the potential of this venom for neutralizing HIV virions, possibly by affecting the viral envelope protein or interfering in the fusion process.

Previous studies have shown that phospholipases (PLA) extracted from bees and snakes could inhibit HIV at an early stage during the viral infection[21,31]. HIV enters the target cells by fusion at the plasma membrane. This process is triggered by the binding of a glycoprotein to CD4 and chemokine receptors on the viral surface. It is interesting that (or it is notable that) unlike PLA isolated from the animal venoms, the human secreted PLA does not show any anti-HIV activity *in vitro*. The previously suggested mechanism for the *in vitro* anti-HIV effect of PLA extracted from *Crotalus durissus terrificus* was the ability of this enzyme to destabilize the fusion complex on HIV target cells[32]. The *H. lepturus* venom was subjected to fractionation to extract the venom PLA and subsequently verify its anti-HIV activity (Figs. 2 and 3). Based on the obtained data, one of the separated PLA from *H. lepturus* had considerable anti-HIV activity (Fig. 4). There is a significant reverse correlation between the anti-HIV and the phospholipase activity of the extracted PLAs, since X1 with the lowest phospholipase activity was the highest inhibitor against the HIV replication. According to our data, the H. *lepturus* venom contains components with the ability to facilitate HIV replication so that a fraction of small peptides (<10 kDa) increased the HIV replication three-fold. Based on these data, the H. *lepturus* venom must contain an anti-HIV component with considerable activity or a certain mode of action that compensates the inducing activity of the low molecular weight (<10 kDa) fraction.

The present study reports the anti-HIV activity of *H. lepturus* venom, which is already known as a hemolytic, cytotoxic, proteolytic and channel blocker agent[9-12]. *H. lepturus* showed neutralizing activity against HIV virions, implying that the active components of this venom interacted with the viral particles or interfered in the fusion process. In this respect, the novel putative anti-viral activity of *H. lepturus* venom will be particularly valuable to be analyzed in the future. The X1 phospholipase of this venom would be a promising candidate in anti-HIV drug discovery programs.
